# Acute Right Ventricular (RV) Failure Presenting As Isolated Right-Sided Colitis

**DOI:** 10.7759/cureus.80427

**Published:** 2025-03-11

**Authors:** Ayham Asassfeh, Ramy Zughul

**Affiliations:** 1 General Practice, Ibn Al Haytham Hospital, Amman, JOR; 2 Critical Care Medicine, University of Michigan, Lansing, USA

**Keywords:** ischemic colitis, localized nomi, nomi, nonocclusive mesenteric ischemia, rv failure

## Abstract

Ischemic colitis constitutes about half of mesenteric vasculopathies, and it shares a similar pathophysiologic process with acute and chronic mesenteric ischemia. These, in turn, can be divided into occlusive and nonocclusive mesenteric ischemia (NOMI). We present this case of a patient presenting with abdominal pain secondary to right-sided colitis, initially presumed to be infectious, but later attributed to localized NOMI. The correlation between localized NOMI, specifically right-sided colitis, and right ventricular (RV) failure has not been well described in the literature. NOMI, in general, is suspected to be secondary to splanchnic vasoconstriction, which results from sympathetic stimulation and renin-angiotensin-aldosterone system (RAAS) activation. Additionally, patients with RV failure have been shown to develop intestinal congestion due to elevated right atrial pressure (RAP), leading to bowel wall thickening and impaired mesenteric venous drainage, which may further contribute to ischemia. This mechanism suggests that both arterial hypoperfusion and venous congestion may play a role in the pathogenesis of right-sided ischemic colitis in RV failure. The predominance of ischemia in the right colon, rather than in classical watershed areas, may be attributed to preferential vasoconstriction of the superior mesenteric artery (SMA), which supplies the right colon, as well as localized venous congestion impairing mesenteric drainage in this region. Individual variability in vascular anatomy and autoregulatory responses could also contribute to this atypical presentation. This case highlights a potentially underrecognized association between RV dysfunction and right-sided ischemic colitis, emphasizing the interplay of low cardiac output, mesenteric hypoperfusion, and venous congestion. Given the absence of direct studies on this relationship, further research is needed to better understand its clinical significance.

## Introduction

Ischemic colitis represents about half of mesenteric vasculopathies and is, in fact, the most common type, with an incidence of ~15 per 100,000 annually. Despite being distinct in classification from acute mesenteric ischemia, which is usually more severe, having a different characteristic presentation and involving the small intestine, ischemic colitis shares similar pathophysiologic processes with acute and chronic mesenteric ischemia. Mesenteric ischemia can be generally divided into occlusive and nonocclusive phenomena (e.g., nonocclusive mesenteric ischemia or NOMI). The latter is a well-known complication of heart disease and is usually caused by decreased cardiac output and hypotension [1,2]. This leads to generalized NOMI resulting from mesenteric arterial vasospasm in response to the circulatory shock. Alternatively, this vasospasm can lead to localized NOMI, usually affecting watershed areas (splenic flexure), and less commonly causing isolated right-sided colitis, or infrequently, isolated cecal colitis, which is rarely recognized in literature. The intestine is particularly vulnerable due to the autoregulatory failure of splanchnic perfusion in prolonged hypotension states, and the right colon, primarily supplied by the superior mesenteric artery, may be affected when systemic circulatory insufficiency reaches critical levels [3].

For instance, in 2006, Cappell, et al. studied 23 patients with myocardial infarction (MI) that developed ischemic colitis, and it was notable that only two of them had right-sided colitis [2]. Although right-sided ischemic colitis is considered uncommon, its occurrence in the setting of right ventricular (RV) failure may be underrecognized. Splanchnic vasoconstriction and reduced mesenteric perfusion in the setting of RV failure may contribute to localized NOMI, providing a potential mechanism for ischemic injury in the right colon. Venous congestion due to RV dysfunction can further impair splanchnic drainage, compounding the ischemic insult, which may help explain why the right colon is vulnerable in this context [3].

Here, we describe a case of isolated right-sided colitis presumed to be the result of localized NOMI in the setting of right heart failure, an under-recognized entity with important clinical implications, where early recognition may aid in timely intervention and improved outcomes.

## Case presentation

History of presenting illness

This is a case of an 81-year-old lady who initially presented to the emergency department (ED) with a complaint of right lower quadrant (RLQ) pain and anorexia. Her past medical history was significant for hypertension, hyperlipidemia, type 2 diabetes mellitus, hypothyroidism, reflux disease and chronic atrial fibrillation, anticoagulated with apixaban, combined left ventricular systolic failure (with left ventricular ejection fraction (LVEF) of 45%) and severe right systolic dysfunction. She reported sharp RLQ pain associated with nausea, without vomiting. She denied having fever or diarrhea, and she denied blood in stool or any urinary symptoms.

Initial Presentation

**Figure 1:**
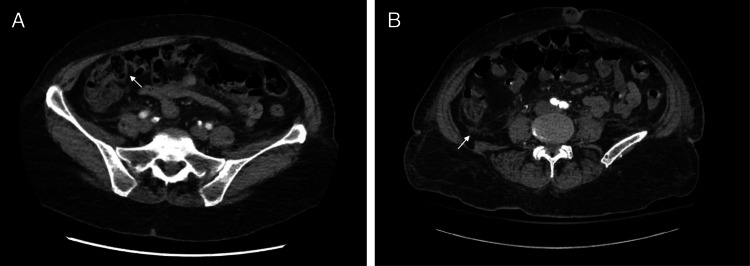
Initial CT abdomen/pelvis with contrast (A) Wall thickening in the ascending colon and terminal ileum, suggestive of ascending colitis. This territory is supplied by superior mesenteric artery. (B) Surrounding fat stranding, which may indicate inflammation or ischemic insult.

On presentation, she was alert and oriented, afebrile and had stable vitals. Physical exam was remarkable for right lower quadrant tenderness without rebound phenomenon, or rigidity, and normal bowel sounds were heard. Chest exam was notable for normal heart sounds and absence of lung crackles or lower extremity edema. Labs showed no leukocytosis, liver function tests (LFTs) were within normal limits, but lactic acid was elevated 3.1 mmol/L (0.5-1.9 mmol/L), and this improved on repeat check after IV hydration. CT abdomen/pelvis with contrast revealed mild wall thickening of the proximal right ascending colon suggestive of colitis, it also showed gallstones without signs of cholecystitis (Figure 1). She was admitted to the floor, her symptoms improved, and she was discharged the next day on oral antibiotics for suspicion of infectious etiology for her colitis.

Second ED visit

She returned to the ED the following day with worsening abdominal pain, this time periumbilical and cramp-like, and nausea, again with no fever nor diarrhea. On exam, she was afebrile, tachycardic (atrial fibrillation), but with stable blood pressure (BP). On physical exam she continued to have RLQ tenderness but no peritoneal signs. Still with no pulmonary congestion or leg edema, exam also noted mild jugular venous distention (JVD).

Investigations

**Figure 2:**
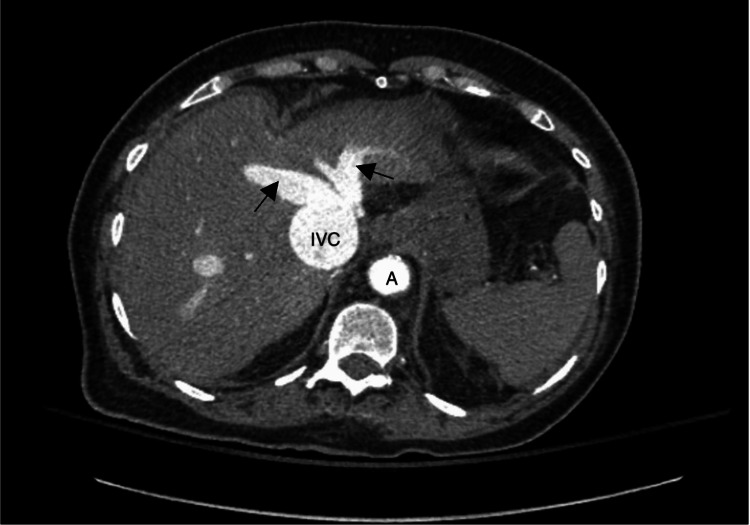
Repeat CT angiogram abdomen/pelvis: hepatic cut Evidence of inferior vena cava (IVC) dilation and contrast backflow into hepatic veins (arrows), consistent with hepatic congestion and elevated right atrial pressure (RAP). A: Aorta.

**Figure 3:**
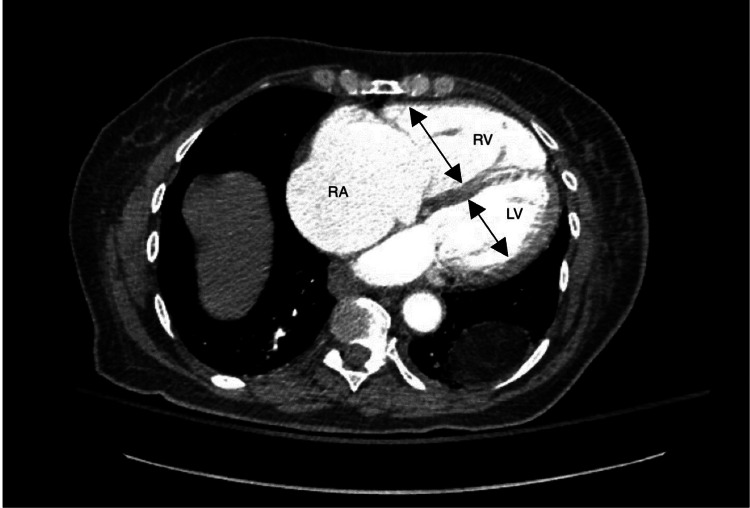
CT angiogram abdomen/pelvis: cardiac cut Marked dilation of the right ventricle (RV) and right atrium (RA), indicative of severe RV dysfunction. Compare with the relatively smaller left ventricle (LV), highlighting the disproportionate right-sided chamber enlargement seen in RV failure.

Labs showed no leukocytosis, routine gastrointestinal polymerase chain reaction (PCR) panel for common pathogens from previous admission was noticed to be negative. Repeat LFTs were initially not obtained, and her lactate was elevated at 5.6 mmol/L. She received 1 liter bolus of fluids with improvement in her heart rate, and BP remained stable. However, her lactic acid continued to be elevated (6.3 mmol/L) even after receiving another liter bolus, so suspicion was raised for ischemic colitis, especially with lack of response to antibiotics. Repeat LFTs came back elevated (Table 1), but with normal total bilirubin (0.7 mg/dL). Baseline LFTs in a prior admission were normal, so transaminitis was considered acute. CT angiography (CTA) abdomen/pelvis was done, which showed patent vessels and improvement in colitis picture (not shown here), with evidence of back flow of hepatic veins and inferior vena cava (IVC) dilation (Figure 2), suggestive of hepatic congestion, and isolated right ventricular and right atrial dilation (Figure 3). The clinical picture became more suggestive of an ischemic etiology that is specifically related to heart failure, given signs of congestive hepatopathy and low output state. The patient was admitted to the intensive care unit (ICU). A repeat echocardiogram showed worsening ejection fraction of 38% and severe RV dysfunction, severe tricuspid regurgitation (TR), and severely elevated right atrial pressure (RAP).

**Table 1:** Lab results on admission

Lab Test	Result	Reference Range
White Blood Cell Count (WBC)	8.2	4.0-11.0 x10^9/L
Lactic Acid	5.6 → 6.3 mmol/L	0.5-1.9 mmol/L
Aspartate Aminotransferase (AST)	500 U/L	10-50 U/L
Alanine Aminotransferase (ALT)	296 U/L	10-50 U/L
Alkaline Phosphatase	150 U/L	32-122 U/L
Total Bilirubin	0.7 mg/dL	0.2-1.0 mg/dL

Intervention

She was started on IV diuretics and the cardiology team was consulted. The gastrointestinal (GI) team was also consulted and recommended a trial of IV N-acetyl-cysteine (NAC). The following day, the patient was transferred to the cardiac care unit (CCU), antibiotics were stopped, a small-dose norepinephrine drip was started for RV support, and she was continued on IV furosemide drip. This was initially intended to increase systemic vascular resistance (SVR) and provide mild inotropic support in the setting of borderline low blood pressures, it was continued without requiring another inotrope after positive response was noticed. The patient remained in the hospital for six more days; lactate cleared and her symptoms improved. Follow-up catheterization on the last day also suggested resolution of critical illness; it was remarkable for mild nonobstructive coronary artery disease (CAD) and showed mildly elevated biventricular pressures. She was eventually discharged from the hospital and advised on close follow-up after discharge.

## Discussion

In this case of isolated ascending colitis with suspected ischemic etiology, CTA of the abdomen and pelvis did not show any signs of vessel obstruction, which supports the suspicion of nonocclusive mesenteric ischemia (NOMI) over other causes of occlusive ischemia.

Among other pathologies, NOMI is reported to occur in low cardiac output states, like congestive heart failure and cardiac surgeries, as well as in hemodialysis patients [4-10]. The hypothesized mechanisms were most recently reviewed by Al-Diery et al. in 2008, who described the possible role of splanchnic vasoconstriction, sympathetic stimulation, renin-angiotensin-aldosterone system (RAAS), endothelin, and prostaglandins in causing intestinal ischemia in shock states [3]. While specific hemodynamic measurements (e.g., cardiac index) were unavailable, the patient’s elevated lactate levels and response to norepinephrine suggest a low-flow state and decreased cardiac output, supporting NOMI as a likely cause. Additionally, lactate clearance with norepinephrine argues against isolated bowel ischemia as the sole cause of lactic acidosis, and suggests a cardiac-related etiology. The absence of evidence of mesenteric occlusion on CTA effectively rules out embolic phenomenon. We suspect severe RV failure and tricuspid regurgitation (TR) played a major role in creating a low-output state, possibly as part of biventricular failure with only mild to moderate LV dysfunction. Elevated RAP can impair venous return, leading to splanchnic congestion and contributing to gut ischemia. The combination of venous congestion and low cardiac output likely both played roles in the development of ischemia in this case. However, there is limited literature correlating RV failure with isolated right-sided ischemic colitis, highlighting the need for further research. 

RV failure, often studied in pulmonary hypertension or congenital heart disease (CHD), has been linked to sympathetic nervous system (SNS) overactivation and β1-receptor downregulation, similar to left ventricle (LV) failure. SNS overactivation is thought to preferentially stimulate the RV outflow tract, leading to RV outflow obstruction [11]. However, RV outflow obstruction due to SNS activation is not well-documented in non-structural RV disease and remains speculative. That said, a milder degree of SNS overactivation could still exacerbate RV dysfunction without causing overt obstruction. RV failure is also associated with RAAS activation, though the benefit of RAAS inhibition remains unclear [12]. Overall, SNS activation in this setting may lead to splanchnic vasoconstriction and eventually NOMI.

A study assessing RV dysfunction and bowel function found that patients with elevated RAP had increased bowel wall thickness, suggesting intestinal congestion and reduced stroke volume as possible mechanisms [13]. Intestinal congestion in RV failure results from passive venous stasis, whereas ischemic colitis is driven by arterial hypoperfusion. Both can cause bowel wall thickening, but congestion leads to hydrostatic pressure and interstitial edema, while ischemia triggers inflammation, vascular permeability changes, and mucosal injury. In this case, IVC dilation and hepatic congestion suggest venous stasis contributed to bowel edema, while elevated lactate and improvement with inotropes indicate a component of true systemic ischemia. Mesenteric venous dilation was not explicitly reported, but the presence of systemic congestion supports the likelihood of mixed pathology.

Management of RV failure focuses on addressing underlying causes of decompensation. Preload reduction is crucial when excessive venous return overstretches the RV, though volume administration may be needed if intravascular depletion is suspected, such as in right-sided myocardial infarction. Afterload reduction is also critical, particularly in pulmonary arterial hypertension (PAH), where pulmonary vasodilators may be considered [14]. However, these agents are not approved for RV failure in critical settings and may worsen outcomes in some cases, such as endothelin receptor antagonists, which have increased mortality in LV failure and could negatively impact RV function [15]. When systemic vascular resistance or blood pressure is reduced, vasopressors may be necessary to improve coronary perfusion, particularly when increased pulmonary vascular resistance elevates RV systolic pressures beyond systemic levels. Vasopressor activity in this case should precede agents that increase myocardial contractility [14]. Certainly in some cases, inotropic or mechanical support might be required. The atypical localization of ischemia in the right colon, rather than the classic watershed areas (splenic flexure and rectosigmoid junction), may be due to multiple factors. One possibility is preferential vasoconstriction of the superior mesenteric artery (SMA), which supplies the right colon, potentially driven by variations in autonomic responses, RAAS activation, or individual differences in splanchnic blood flow regulation. Another factor could be venous congestion from elevated RAP, impairing mesenteric drainage and promoting ischemia. While colonic venous drainage is anatomically variable, localized congestion in the right colon may have contributed to its susceptibility in this patient. Studies have demonstrated significant variability in ascending colon venous anatomy, with the right colic vein (RCV) absent in a large percentage of individuals, necessitating alternative drainage pathways. These variations may influence the degree of congestion in different colonic segments and could have played a role in this case [16]. This suggests that the patient’s right-sided colitis resulted from a combination of arterial hypoperfusion and venous congestion, rather than purely a vasoconstrictive NOMI process. Recognizing this atypical localization highlights the complexity of RV failure-induced mesenteric ischemia, which may not always follow classic NOMI patterns.

Beyond its established role in treating acetaminophen-induced liver failure, NAC has demonstrated potential benefits in non-acetaminophen-related acute liver failure, including cases of ischemic hepatopathy. Its mechanisms include replenishing glutathione stores, scavenging free radicals, and enhancing microcirculatory blood flow, thereby mitigating oxidative stress and improving hepatocellular function [17-20].

## Conclusions

Ischemic colitis results from decreased perfusion of the colonic wall and mesentery. While mesenteric ischemia is often attributed to vascular occlusion from atherosclerosis or embolic phenomena, we suspect that a low-output state and venous congestion from RV systolic dysfunction contributed in this case. Though direct evidence linking RV failure to isolated right-sided colitis is lacking, the interplay of reduced mesenteric perfusion, splanchnic vasoconstriction, and venous congestion may predispose to ischemia in this region. Given the absence of studies on this relationship, further research is warranted.
